# Epidemiology of Motoric Cognitive Risk Syndrome in the Kerala Einstein Study: Protocol for a Prospective Cohort Study

**DOI:** 10.2196/49933

**Published:** 2023-08-17

**Authors:** Sanish Sathyan, Emmeline Ayers, Helena Blumen, Erica F Weiss, Dristi Adhikari, Marnina Stimmel, Kizhakkaniyakath Abdulsalam, Mohan Noone, Roy K George, Mirnova Ceide, Anne Felicia Ambrose, Cuiling Wang, Poornima Narayanan, Sachin Sureshbabu, Kunnukatil S Shaji, Alben Sigamani, Pavagada S Mathuranath, Vayyattu G Pradeep, Joe Verghese

**Affiliations:** 1 Department of Neurology Albert Einstein College of Medicine Bronx, NY United States; 2 Department of Medicine Albert Einstein College of Medicine Bronx, NY United States; 3 Institute of Neurosciences Baby Memorial Hospital Kozhikode India; 4 Department of Psychiatry and Behavioral Sciences Montefiore Medical Center Albert Einstein College of Medicine Bronx, NY United States; 5 Department of Epidemiology and Population Health Albert Einstein College of Medicine Bronx, NY United States; 6 Centre for Neurosciences Meitra Hospital Kozhikode India; 7 Kerala University of Health Sciences Thrissur India; 8 Carmel Research Consultancy Pvt Ltd Bengaluru India; 9 Department of Neurology National Institute of Mental Health and Neurosciences Bengaluru India

**Keywords:** motoric cognitive risk, Kerala, India, dementia, cognitive decline, neuroimaging

## Abstract

**Background:**

The southern India state of Kerala has among the highest proportion of older adults in its population in the country. An increase in chronic age-related diseases such as dementia is expected in the older Kerala population. Identifying older individuals early in the course of cognitive decline offers the best hope of introducing preventive measures early and planning management. However, the epidemiology and pathogenesis of predementia syndromes at the early stages of cognitive decline in older adults are not well established in India.

**Objective:**

The Kerala Einstein Study (KES) is a community-based cohort study that was established in 2008 and is based in the Kozhikode district in Kerala state. KES aims to establish risk factors and brain substrates of motoric cognitive risk syndrome (MCR), a predementia syndrome characterized by the presence of slow gait and subjective cognitive concerns in individuals without dementia or disability. This protocol describes the study design and procedures for this KES project.

**Methods:**

KES is proposing to enroll a sample of 1000 adults ≥60 years old from urban and rural areas in the Kozhikode district of Kerala state: 200 recruited in the previous phase of KES and 800 new participants to be recruited in this project. MCR is the cognitive phenotype of primary interest. The associations between previously established risk factors for dementia as well as novel risk factors (apathy and traumatic brain injury) and MCR will be examined in KES. Risk factor profiles for MCR will be compared between urban and rural residents as well as with individuals who meet the criteria for mild cognitive impairment (MCI). Cognitive and physical function, medical history and medications, sociodemographic characteristics, lifestyle patterns, and activities of daily living will be evaluated. Participants will also undergo magnetic resonance imaging and electrocardiogram investigations. Longitudinal follow-up is planned in a subset of participants as a prelude to future longitudinal studies.

**Results:**

KES (2R01AG039330-07) was funded by the US National Institutes of Health in September 2019 and received approval from the Indian Medical Council of Research to start the study in June 2021. We had recruited 433 new participants from urban and rural sites in Kozhikode as of May 2023: 41.1% (178/433) women, 67.7% (293/433) rural residents, and 13.4% (58/433) MCR cases. Enrollment is actively ongoing at all the KES recruitment sites.

**Conclusions:**

KES will provide new insights into risk factors and brain substrates associated with MCR in India and will help guide future development of regionally specific preventive interventions for dementia.

**International Registered Report Identifier (IRRID):**

DERR1-10.2196/49933

## Introduction

### Background

India’s older adult population (≥60 years old) currently is comprised of 146 million people and is expected to increase to 179 million people by 2030 [1]. Southern India (including the state of Kerala) has the highest number of older adults—one-quarter of India’s older adults [2]. Kerala has had the highest life expectancy rates since the 1970s when compared with all other Indian states (Kerala: 75.2 years; India: 69.7 years) [3]. Kerala has the highest prevalence of older adults (16.5%) among all Indian states, and this number is projected to increase to 20.9% by 2031 [4]. High literacy rates, access to health care, and a relatively strong public health system in Kerala have been hypothesized to play important roles in this demographic profile.

The increase in the older adult population in Kerala is expected to be accompanied by an attendant increase in chronic age-related diseases, including dementia [5]. Only a few studies have examined the prevalence of dementia in Kerala, with prevalence rates ranging from 3.2% to 8.7% [6-9]. A dementia prevalence of 4.9% was reported in adults aged 55 years and older in Thiruvananthapuram, the capital city of Kerala [6]. Independent studies carried out in urban and rural areas of Ernakulam district in Kerala reported dementia prevalence rates of 3.36% (≥65 years old) and 3.39% (≥60 years old), respectively [7,8]. The Harmonized Diagnostic Assessment of Dementia for the Longitudinal Aging Study in India, the first cross-national study of dementia in India, reported higher dementia prevalence rates of 7.8% and 8.7% in urban and rural areas in Kerala, respectively [9]. A 10-year follow-up study in Kerala reported incidence rates per 1000 person-years for Alzheimer disease of 11.67 (95% CI 10.9-12.4) for those aged ≥55 years and 15.54 (95% CI 14.6-16.5) for those aged ≥65 years [10].

Recognizing the need for a comprehensive study of cognitive decline in this population, the Kerala Einstein Study (KES) was established in 2008 to understand the epidemiology of and risk factors for cognitive decline, Alzheimer disease, and related dementias [11-13]. The primary phase of recruitment of this study spanned from 2008 to 2016. During this initial phase, we successfully developed a clinical research center in Baby Memorial Hospital (BMH) [14] in Kozhikode City in Kerala, validated culture-fair cognitive tests, identified new dementia risk factors (eg, depression, polypharmacy, cardiovascular disease, and small vessel disease [SVD]) [12,15-17], and established magnetic resonance imaging (MRI) protocols and processing pipelines [18,19]. KES (2R01AG039330-07) was renewed by the National Institutes of Health in September 2019 and received approval from the Indian Council of Medical Research (ICMR) to start the study in June 2021.

**Figure 1:**
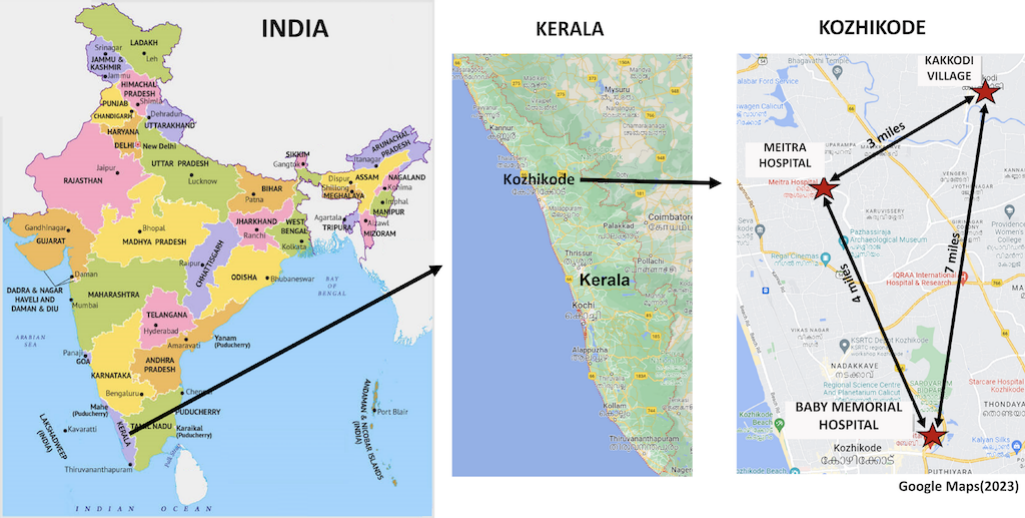
Kerala Einstein Study research site in Kozhikode, Kerala, India.

In this KES project, we target risk factors and brain substrates of predementia syndromes, especially the motoric cognitive risk syndrome (MCR). MCR is a predementia syndrome characterized by cognitive complaints and slow gait, proposed by Verghese and colleagues in 2013 [20], and validated in multiple countries [13,21-23]. MCR predicts both Alzheimer disease and vascular dementia even after accounting for overlap with mild cognitive impairment (MCI) [13,20,21]. MCR is common; the prevalence was 9.7% in our 17-country study (including KES) [13]. Unlike MCI, complex cognitive tests or assays are not needed to diagnose MCR [13,20,21], increasing its clinical utility in resource-poor low- and middle-income countries (LMIC) like India. We have identified several potentially modifiable risk factors (depression, sedentary lifestyle, and obesity) for MCR in high-income countries [21], but their association with MCR in LMIC is unknown. In this KES project, we will study 2 novel risk factors for predementia syndromes in KES: apathy and mild traumatic brain injury (TBI). Recent investigations highlighted apathy as a separate entity from depression and uniquely associated with cognitive decline [13]. Although TBI is a leading cause of disability in LMIC, its role as a contributor to late-life cognitive decline in LMIC is underrecognized. We hypothesize that apathy and TBI will be associated with MCR and that these associations will be moderated by early-life (years of schooling) and late-life participation in cognitive activities. We also aim to explore commonalities and differences in risk factors for predementia syndromes such as MCR in urban (Kozhikode City) and rural (Kakkodi village) communities in Kozhikode district, Kerala (Figure 1).

**Figure 2:**
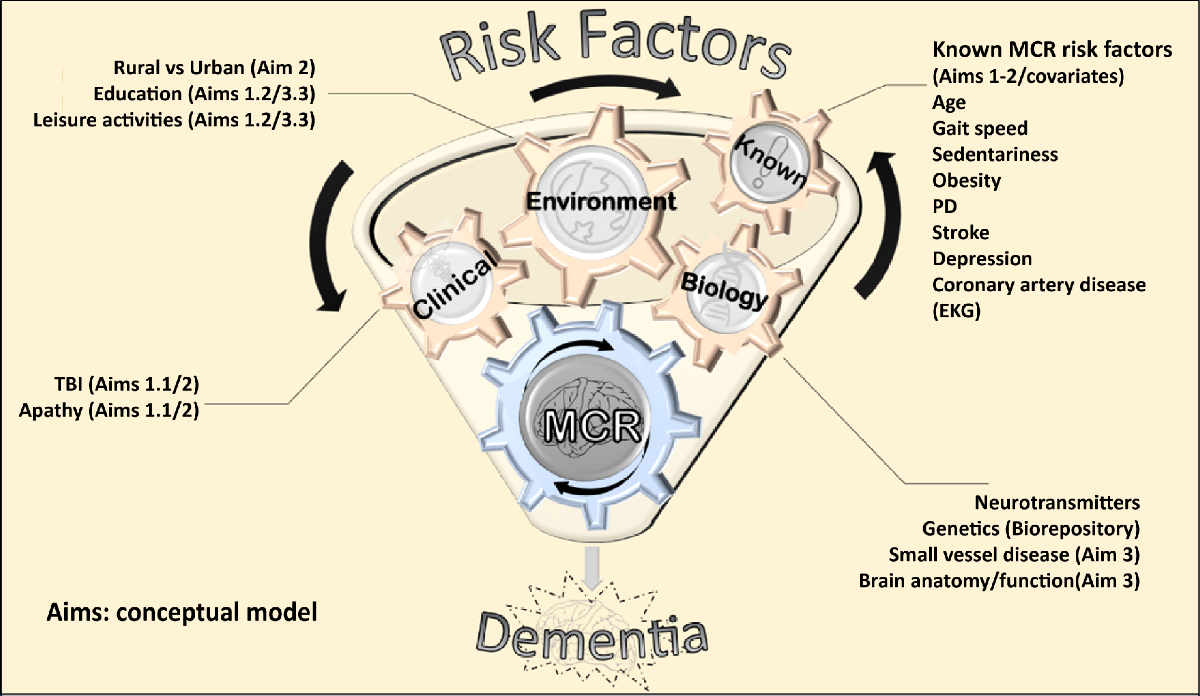
Conceptual framework of the Kerala Einstein Study. EKG: electrocardiogram; MCR: motoric cognitive risk syndrome; PD: Parkinson disease; TBI: traumatic brain injury.

Figure 2 shows the conceptual framework of the KES. This study will identify relationships between different risk and protective factors for MCR in this southern Indian population. This study will have a major impact on building sustainable research capacity in this region. The extensive data collected as part of this study will act as a rich resource for discovery as well as validation of risk factors of other age-associated phenotypes and syndromes.

### Study Aims

The main aims of this observational study are threefold. First, the study aims to examine clinical risk factors for predementia syndromes (MCR and MCI) in 1000 older adults from Kerala: 200 already recruited in phase 1 of KES and 800 new recruits from urban and rural sites (Aim 1). The following will be conducted to support this aim:

- Examine the association of potentially modifiable risk factors previously identified in high-income countries as well as novel risk factors (apathy and TBI) with MCR and MCI in 1000 Kerala seniors (Aim 1.1)
- Examine if the relationship between apathy or TBI and MCR or MCI is moderated by early-life (years of schooling) and late-life participation in cognitive activities (Aim 1.2)

Second, we will explore commonalities and differences in risk factors for predementia syndromes in rural and urban Kerala older adults (Aim 2). Third, we will establish brain substrates and pathologies of MCR in Kerala older adults (Aim 3). The following activities will support this aim:

- Examine structural brain abnormalities (gray matter atrophy, reduced structural connectivity) and SVD pathologies in adults with MCR compared with healthy aging controls (Aim 3.1)
- Examine the relationship between structural brain abnormalities and MCR and the role of SVD in mediating this association (Aim 3.2)
- Examine the impact of early-life and late-life participation in cognitive activities on the association between structural brain abnormalities and MCR (Aim 3.3)

## Methods

### Study Design and Population

We propose a cross-sectional survey of 1000 older adults in urban and rural locations in Kozhikode district. We also propose follow-up assessments at 12-month intervals with participants at our rural site. This will be the first epidemiological survey of predementia syndromes from this region and will provide preliminary data to develop new biological hypotheses. The KES project is based in Kozhikode, a northern district in Kerala state with a metropolitan population of more than 2 million people, making it the 19th largest urban agglomeration in India. The initial phase (2008-2016) of the KES was based in BMH—one of the leading providers of medical care in Kozhikode City [14]. Participants (n=200) were identified from patients and caregivers attending the neurology clinic at BMH.

An additional 800 new participants will be recruited in this phase of KES. Brain MRI will be performed with 400 participants (180 completed in the initial phase of KES and 220 new studies in this project), and up to 154 repeat MRIs will be obtained at 12-month intervals. Large-scale neuroimaging investigations are limited in India. The KES will create a unique MRI database to examine brain substrates of cognitive decline in this population.

A new component included in this KES study is the addition of rural and exurban recruitment sites in Kozhikode district. The rural site will be in Kakkodi village (population: 42,866 people) [24]. Kakkodi village differs from Kozhikode City in several indicators such as socioeconomic status (lower), occupational types (agricultural and household work are the major occupations), health care access (no hospitals nor specialty clinics), educational institutions (only primary school and middle school), and transportation (limited bus service and no trains). These rural-urban differences disproportionately affect rural-dwelling older adults who may be less mobile. There are no hospitals in Kakkodi, but BMH maintains a community health center where 500 to 600 Kakkodi adult residents receive routine medical care annually. Patients with more acute or serious complaints are referred to BMH and other hospitals in Kozhikode City. We will utilize this BMH community health center to recruit rural-dwelling older adults. The second new site will be Meitra Hospital [25], located in an exurban (beyond the suburbs) location outside Kozhikode city. As in BMH, potential participants will include patients attending neurology and other specialty clinics as well as older caregivers or family members who meet KES eligibility criteria. Meitra Hospital is a multidisciplinary institution with outpatient and inpatient facilities. Although BMH and Meitra Hospital are in urban and exurban locations, respectively, the patient population in both hospitals includes a mix of urban and rural patients who are referred from all over Kozhikode district as well as neighboring districts. We will track urban or rural residency for our analyses (regardless of study site) based on participants’ home addresses.

### Inclusion and Exclusion Criteria

Inclusion criteria are (1) age of 60 years or older and (2) being able to provide informed consent. Exclusion criteria are (1) refusal to complete the study tasks (ie, we had <5% refusal rate in the initial KES phase), (2) previous physician-diagnosed dementia, and (3) presence of acute or terminal illnesses or (4) presence of severe auditory or visual loss that would make it difficult for the participant to complete cognitive tasks. In addition, for neuroimaging studies, standard exclusion criteria apply, such as presence of pacemakers; aneurysm clips; artificial heart valves; ear implants; and metal fragments or foreign objects in the eyes, skin, or body.

### Data Collection and Measures

Study protocols were collaboratively developed by the KES team in India and the United States over the past decade. Study procedures, testers, and supervision will be uniform and harmonized across all 3 field centers in Kozhikode district. Several culturally appropriate strategies approved by the local institutional review board (IRB) are in place to optimize recruitment and retention. For instance, all testing will be administered in Malayalam (local language) or English, as preferred by participants [11,26-28]. Results of electrocardiogram (ECG) and neuroimaging studies will be provided to the participants to share with their medical providers.

Once participants provide informed consent, they and their caregiver, if appropriate, will complete a standardized interview with the research assistant (RA) to obtain demographic characteristics, comprehensive medical history including TBI history, current medications, functional status, and mood states. Participants also complete robust measures of cognitive and physical functioning and undergo ECGs. Those who consent for the neuroimaging substudy will also complete MRIs of the brain. KES assessments are described in detail in the following sections.

### Cognitive Tests and Self-reported Measures

Neuropsychological measures will be completed to evaluate cognitive function and to diagnose cognitive impairment or dementia in this cohort. Table 1 provides a catalog of the neuropsychological tasks conducted in KES. All KES tests have established Malayalam and English versions.

**Table 1:** Tests conducted in the Kerala Einstein Study—local language (Malayalam) versions of all tests are available.

Category and measure		Instrument(s)
**Cognition**		
	General	Addenbrooke Cognitive Examination (ACE) [26,28]
	Memory	Picture-based memory impairment screen [11] Verbal Learning Test [29,30] Modified Taylor Complex Figure recall [29-31]
	Attention and executive function	Digit Symbol Substitution Test [32] Verbal fluency [27,29] Trail Making Test-Black and White [29,30,33] WMS-R^a^ Digit Span [26,34]
	Visuospatial	Modified Taylor Complex Figure [29,31]
Mood		Geriatric Depression Scale-15 [35] Generalized Anxiety Disorder-7 [36]
**Function**		
	ADL^b^	Kerala ADL scale [37]: Instrumental/basic
**Social**		
	Network support	Social Network Index [38] Social Support Survey [39]
**Illness**		
	Comorbid index	Charlson Comorbid Index [40] Clinical interview
**Vascular**		
	Coronary artery disease	Electrocardiogram [12,41] Clinical interview

^a^WMS-R: Wechsler Memory Scale-Revised.

^b^ADL: activities of daily living.

The Addenbrooke Cognitive Examination (ACE) [28] and picture-based memory impairment screen [11] will be used to provide estimates of global cognition and memory function, respectively. For those participants who agree to complete more comprehensive testing, neuropsychological measures will include those chosen from the ICMR-Neurocognitive Toolbox (NCTB), a validated battery created in India and translated into various Indian languages including Malayalam [42]. The following ICMR-NCTB measures will be selected: Verbal Learning Test (verbal learning and memory), Modified Taylor Complex Figure Test (visuospatial and visual memory), Trail Making Test Black and White (A and B; visuospatial attention, cognitive speed, and set-shifting), Category Fluency Test (verbal fluency), and Phonemic Fluency Test (verbal fluency). The Wechsler Memory Scale-Revised Digit Span Task, which was previously used and validated in Kerala, will be used to assess auditory attention and working memory [43].

Participants will complete validated self-report questionnaires assessing depressive symptoms (Geriatric Depression Scale-30 [GDS-30] [42]), anxiety (Generalized Anxiety Disorder [GAD-7] [36]), apathy (Apathy Evaluation Scale [AES] by Marin et al [44]), social networks (Social Network Index [45] and Medical Outcomes Study Social Support Survey [46]), happiness (Subjective Happiness Scale [39]), and current leisure activities (Leisure Activity Scale [47]).

Level of independent functioning will be measured using the Instrumental Activities of Daily Living Scale for elderly people, which was developed in Kerala as a scale of functional abilities [37]. Subjective cognitive complaints will be operationalized using the 20-item Cognitive Change Index [48] and Subjective Cognitive and Motoric Complaint Questionnaire**.** The latter instrument was developed by our team to ascertain participant concerns regarding their cognition and motoric function.

All cognitive tasks and questionnaires will be administered by KES RAs, trained and supervised by the KES clinical neuropsychologists (EFW and MS). RAs in Kerala are trained in the administration and scoring of neuropsychological measures via televideo by a licensed clinical neuropsychologist (MS) based in the United States. Training includes review and explanation of each of the neuropsychological measures, modeling of test administration, and practice administration and scoring. RAs will be cleared by the neuropsychologist (MS) to evaluate participants following acceptable televideo practice administration. Quality control measures include regular meetings with the neuropsychologist (MS) and RAs to refresh and review procedures as well as periodic review of scored protocols by the neuropsychologist. In addition, all study protocols are double scored by a single dedicated RA (who did not administer the tests) to ensure consistency in scoring across RAs and to identify and correct any observed errors in administration and/or scoring procedures.

### Physical Tests

#### Gait

Timed walks at a normal pace over a fixed distance (10 feet) will be completed by participants. Use of any walking aid will be noted. We have reported that older adults with MCI had slower walking times compared with cognitive normal controls in KES [49].

#### Unipedal Stance

The unipedal stance test is a widely used clinical test of balance. Participants will be timed as they stand on 1 leg (participant will choose which leg) for a maximum of 30 seconds [50]. “Failure” is defined as shifting the stance foot or placing the lifted foot on the floor [50]. The test is stopped and considered normal at 30 seconds*.*

#### Grip Strength

A Jamar handgrip dynamometer will be used to objectively measure grip strength as the maximum voluntary contraction in the dominant hand. Grip strength is used to define frailty and is a validated marker of physical function [51].

#### Repeated Chair Rise

To test leg strength and endurance, participants will perform the repeated chair rise test using a standard padded chair [52]. Participants are instructed to stand and sit down on the chair 5 times as quickly as they can with their arms folded across their chest. Time taken to complete the 5 trials will be recorded.

### Neuroimaging: MRI Protocol and Assessment

#### Image Acquisition

##### Overview

All images are acquired on a 3T Siemens MAGNATOM-Skyra at Meitra Hospital. T1-weighted whole head structural images are acquired using axial 3D-MP-RAGE parameters over a 256 mm field of view (FOV) and 1.0 mm isotropic resolution (echo time [TE]=2.26 ms, repetition time [TR]=2300 ms, α=80). Diffusion-weighted images are acquired at b=1000 s/mm^2^ for 64 gradient directions. In addition, 6 b=0 s/mm^2^ volumes are collected, one-half with opposing phase encoding directions to correct for distortions. 3D fluid-attenuated inversion recovery (FLAIR) images are acquired over a 256 mm FOV with 0.89 mm resolution (TE=387 ms, TR=4,500 ms, inversion time=1800 ms). Finally, 3D-susceptibility-weighted images (SWIs) are acquired over 216x256 FOV with a 0.86 mm resolution (TE=20 ms, TR=27 ms). The Meitra Hospital neuroradiologist reviews each MRI scan. Any significant neuroradiological abnormality is communicated to the KES neurologists by the neuroradiologist for consideration of further action. The MRI reports are shared with the participant. Each MRI examination takes approximately 1 hour including safety screening, set up, and scanning.

##### Gray Matter Volume and Cortical Thickness: Preprocessing

Parcellation of the T1-weighted data into anatomical brain regions is important for examining gray matter volume and cortical thickness patterns associated with MCR and is a necessary step in processing data for examining structural connectivity associated with MCR. Each participant’s structural T1-weighted image will be reconstructed using FreeSurfer v 7.2 [53]. The accuracy of FreeSurfer’s subcortical segmentation and cortical parcellation has been reported to be comparable to manual labeling [54]. Gray matter segmentation and longitudinal alignment will be performed with the FreeSurfer longitudinal pipeline [53], which is robust to initialization points and avoids biasing toward any one time point by generating a median template for each participant based on the T1-weighted images at both time points using cubic spline interpolation in FreeSurfer [55]. Several processing steps, such as skull stripping, Talairach transforms, and atlas registration as well as spherical surface maps and parcellations will then be initialized with common information from the participant-specific template, significantly increasing reliability and statistical power. The gray matter volumes of 68 different regions extracted from the FreeSurfer processing pipeline are entered in subsequent statistical analyses.

##### Structural Connectivity: Preprocessing

Preprocessing of diffusion-weighted images consists of field distortion correction using topup distributed as part of the Functional Magnetic Resonance Imaging of the Brain Software Library (FSL; [56]) and eddy current and movement correction using the eddy tool in FSL. To evaluate any white matter changes as a function of MCR status, we will use Tracts Constrained by Underlying Anatomy (TRACULA) distributed as part of the FreeSurfer v 7.2 library. TRACULA uses probabilistic tractography to extract 42 major white matter tracts. The software performs informed automatic tractography by incorporating anatomical information from a training data set, provided by the software, with the anatomical segmentation of the T1 image of the current data set, thus increasing the accuracy of the white matter tract placement for each participant by incorporating each participant’s anatomical data into the tractography algorithm. Parcellation results from the FreeSurfer longitudinal stream will be applied to TRACULA to increase sensitivity to longitudinal changes in white matter tracts. The software outputs white matter integrity measures for each voxel inside the 42 tracts with a mean of about 500 voxels per tract. Thus, for each participant, voxel-wise white matter integrity measures for 42 tracts at baseline and follow-up will be used to test the association with MCR.

Lacunes with presumed vascular origin are defined (consistent with recent consensus criteria) as round or ovoid, subcortical, fluid-filled cavity, 3-15 mm diameter, with a surrounding rim [57]. Infarcts located in white matter must be hypointense on T1-weighted and FLAIR images to distinguish from white matter hyperintensities (WMH).

WMH are automatically quantified from 3D FLAIR images using the lesion segmentation toolbox [58], which will be implemented with SPM12/MatLab [59]. The lesion segmentation tool pipeline provides us with overall WMH lesion count and volume, as well as a voxel-based WMH probability map that can be entered into subsequent analyses.

Microbleeds are identified on SWI, a 3D T2* sequence attuned to detecting hemorrhage [60-63]. SWI was shown to detect 67% more microbleeds than conventional T2 [64,65]. Longitudinal tracking of microbleeds on SWI correlates with cognitive decline [64,65]. We will use the Microbleed Anatomic Rating Scale (MARS) to quantify microbleeds by number and location on SWI [66]. The MARS quantifies microbleeds in the following 9 regions: frontal, parietal, temporal, occipital, infratentorial (brain stem and cerebellum), basal ganglia, thalamus, capsule, and corpus callosum. Regional and overall number and presence of microbleeds will be examined. MARS was used in our MCR study based in KES.

### ECG

A standard 12-lead ECG (Tricog Health) will be performed by a certified cardiac physiologist at BMH and Meitra Hospital [12,41]. Standard 12-lead ECG will be analyzed following the Minnesota code classification by a study clinician, blinded to neurological status including normal, MCR, or dementia diagnoses. Based on our previous KES projects, the following abnormalities will be reported: rhythm abnormalities, left ventricular hypertrophy, significant ST and T wave changes, Q waves, heart block, and bundle branch block [12]. Major ECG abnormalities are defined as the presence of any one of the following: Q-QS wave abnormalities, left ventricular hypertrophy, Wolff-Parkinson-White syndrome, complete bundle branch block or intraventricular block, atrial fibrillation or atrial flutter, or major ST-T changes.

### Phenotypes of Interest

#### MCR

MCR is our primary outcome of interest and is algorithmically diagnosed using the criteria by Verghese et al [13,20,21] in KES participants who meet all 4 of the following criteria: (1) subjective cognitive complaint, (2) slow gait, (3) no functional decline, and (4) no dementia [13,20]. Assessment of the key MCR criteria is summarized in the following paragraphs.

Subjective cognitive complaints are assessed based on participant positive (abnormal) responses to standardized questions in the Subjective Cognitive and Motoric Complaint Questionnaire [67].

Consistent with prior MCR studies, including KES [13,21-23,68,69], slow gait is defined as a gait speed 1 SD below age and sex-specific means. Although gait is multifactorial with neurological and non-neurological causes (eg, arthritis) [70], multiple studies have shown that, irrespective of causes, slow gait predicts dementia, which supports the robustness of this sign [71,72].

#### MCI

We have implemented the following established criteria for MCI [73] in the KES [15,16,18]: (1) score 1.5 SD below age and education-adjusted means on any cognitive test (Table 1), (2) cognitive complaints, (3) no functional decline [37], and (4) no dementia.

#### Dementia

Diagnosis of dementia is made by consensus of study clinicians (neurologist and neuropsychologists) and research staff following review of all available clinical evaluations, neuropsychological test results, functional assessments, and medical history. The Diagnostic and Statistical Manual of Mental Disorders IV (DSM-IV) criteria for diagnosis of dementia will be used [74]. Dementia at baseline is an exclusion criterion for the KES.

#### TBI

Mild TBI is being examined as a novel predictor of MCR in the Kerala population. We will use the American Congress of Rehabilitation Medicine definition of mild TBI that is widely used in clinical and research practice [75]. Mild TBI results in ≤30 minutes of unconsciousness (cf >30 minutes in moderate/severe TBI), and the posttraumatic amnesia period is <24 hours. We will use a local adaptation of the Brain Injury Screening Questionnaire (BISQ) [76], a structured questionnaire that characterizes incidence and severity of lifetime exposure to head trauma and TBI [77-79]. The BISQ provides a profile of symptoms in the following 4 domains: attention and memory; depression, anxiety, and mood; aggression and impulsivity; and physical [78,79]. The BISQ may be completed via interview or self-administration. The BISQ has good-to-excellent test-retest reliability [76,78,79].

#### Apathy and Depressive Symptoms

Apathy has been reported to predict risk of developing predementia syndromes including MCR in the United States [80,81]. We will measure depression and apathy utilizing the GDS-30 [25] and the AES, respectively [44]. Confirmatory factor analysis has shown apathy-withdrawal to be a distinct domain of the GDS [82]. The GDS-3A is the apathy subscale of the GDS, consisting of the following 3 items (score range 0-3) [83]: (1) “Have you dropped many of your activities and interests?” (2) “Do you prefer to stay at home, rather than going out and doing new things?” and (3) “Do you feel full of energy?” A score of 2 or more indicates presence of apathy. Reported sensitivity and specificity of the GDS-3A varied from 29% to 69% and from 89% to 93%, respectively, in 3 large European cohorts of older adults [83,84]. GDS-3A has been used to measure apathy in our Bronx-based cohorts [85]**.** The AES [44], an 18-item questionnaire, assesses apathy symptom severity and 3 domains of apathy (behavioral, emotional, and cognitive). AES scores range from 18 to 72, with higher scores indicating greater severity [44]. The AES has been reported to have higher sensitivity than the GDS-3A, as well as excellent test-retest and inter-rater reliability [44,86]. The AES is recommended for evaluation of people with dementia [87,88] but has not been widely used in aging cohorts [44]. We will develop a local language version of the AES and pilot it in our new sites as an alternate apathy assessment. As the GDS-30 is more widely used, we will also use confirmatory factor analysis of the GDS-30 to partition out apathy from depression in order to develop a more robust measurement model, which we will compare with the AES.

### Statistical Analysis

#### Outcomes

The primary outcome of interest is MCR (and MCI) status. Predictors are continuous and categorical measures including apathy, mild TBI, cognitive reserve (education and leisure activities), and neuroimaging variables. We will examine modifiable predictors for MCR (identified in the United States) such as obesity and sedentariness [21] as well as novel risk factors that we associated with the presence of cognitive impairment or MCI in KES such as ECG abnormalities, apathy, and TBI [12,41]. Baseline distribution of variables will be examined using appropriate graphical procedures and summary statistics before analyses. Transformation of continuous biomarkers will be considered if severe skewness in distributions is detected. We will also address sex in our analyses, both adjusting for it as a covariate and conducting stratified analyses by sex. Cross-sectional associations between both established and novel predictors and MCR/MCI will be evaluated using logistic regression models. All models will adjust for age, gender, education, and other confounders including but not limited to medical illnesses, anthropometric measures, residency status, recruitment source, and global cognition (ACE score). We will compare risk factors for MCR/MCI between urban and rural older adults using logistic regression models in the overall sample as well as separately.

Gray matter volumes and cortical thickness of 84 brain regions will be extracted from the FreeSurfer processing pipeline and entered in linear mixed effects models (LMEMs) adjusted by standard covariates (age, sex, education, total intracranial volume, global health score, and MCI) to detect differences by MCR status. To evaluate white matter integrity or structural connectivity associated with MCR, voxel-wise white matter integrity—fractional anisotropy—measures for 42 tracts at each time point will be entered into LMEMs adjusted by standard covariates (age, sex, education, total intracranial volume, and global health score) to detect differences by MCR status. Finally, the influence of SVD in general as well as specific SVD pathologies (lacunes, WMH, and microbleeds) will also be examined in subsequent LMEMs adjusted by standard covariates (age, sex, education, total intracranial volume, and global health score) to detect differences by MCR and non-MCR status.

#### Power

With a proposed sample size of 1000 and estimated MCR prevalence of 15% [13], we can detect an odds ratio of 1.33, corresponding to a 1 SD increase in worsening apathy and TBI, with 80% power at a significance level of .05. Prevalences of modifiable risk factors for MCR in KES ranges from 10% to 70%, which allows a detection of odds ratios of 1.69 to 2.24 on the associations of these risk factors with MCR and MCI with 80% power [15,16,18].

#### Power (Neuroimaging)

With 400 participants with neuroimaging measures and a prevalence of 15% for MCR or MCI, we can detect a difference of 0.45 SD in structural brain abnormalities between participants with and without MCR or MCI with 80% power. For comparison, we derived a structural brain network of MCR in 267 participants [19].

### Timeline

Over the 5 years of this project, we will seek to recruit approximately 400 urban-dwelling and 400 rural-dwelling participants. Of the 800 new participants, 220 from all sites will undergo neuroimaging, which, along with the 180 studies from the first KES phase, will provide a database of 400 neuroimaging studies. Participants from all 3 sites will be recruited for imaging studies. Given the restricted geographic location of our rural site (Kakkodi village) and very low rates of out-migration by older residents, we propose to follow up rural participants every 12 months. In our urban sites, we will attempt to conduct 12-month follow-up assessments with all participants undergoing neuroimaging. Given the larger catchment area, follow-up will be more challenging, but we will also consider home assessments to improve retention.

### Ethics and Procedures

This study will be conducted in accordance with the Helsinki Declaration. The informed consent and recruitment processes follow local socially and culturally appropriate practices. The assessments and procedures have been approved by the IRB at BMH (BMH/IEC/02/2022), which also served as the local IRB for the first phase of KES. In addition, study design and consent procedures have also been approved by the ICMR (2020-10058), which is a requirement for epidemiological and experimental studies conducted in India. The study protocols have also been reviewed and approved by the Albert Einstein College of Medicine IRB (2006-368).

## Results

We had recruited 433 participants as of May 17, 2023. The basic characteristics of the recruited participants at each of the research sites in Kozhikode are shown in Table 2. As the recruitment is ongoing, formal statistical group comparisons were not conducted. Most of the recruited participants are men (255/433, 58.9%), as noted in previous KES publications [12,13,15,16,18,49]. Most participants (293/433, 67.6%) recruited thus far, including those from BMH and Meitra Hospital, have rural residency. Of the 433 participants recruited thus far, 58 (13.4%) met the criteria for MCR, and 89 (20.6%) met the criteria for MCI.

**Table 2:** Demographic characteristics of participants recruited in the Kerala Einstein Study as of May 17, 2023.

Characteristics		Total (N=433)	BMH^a^ (n=194)	Mietra Hospital (n=102)	Kakkodi (n=137)
Age (years), mean (SD)		68.89 (5.48)	68.46 (5.24)	70.89 (5.22)	68.02 (5.66)
Sex (female), n (%)		178 (41.1)	81 (41.8)	30 (29.4)	67 (48.9)
**Residence, n (%)**					
	Rural	293 (67.6)	107 (54.9)	61 (59.8)	125 (91.2)
	Urban	137 (31.7)	87 (44.6)	39 (38.2)	12 (8.8)
Education (years), mean (SD)		9.20 (3.65)	9.35 (3.76)	9.91 (3.93)	8.47 (3.13)
Addenbrooke Cognitive Examination (ACE) score, (range 0-100), mean (SD)		78.37 (13.07)	77.56 (14.73)	77.58 (11.42)	80.10 (11.51)
Picture-based Memory Impairment Screen (range 0-8), mean (SD)		6.95 (1.90)	6.94 (1.98)	6.12 (2.36)	7.59 (0.94)
Geriatric Depression Scale (range 0-30), mean (SD)		7.76 (6.06)	7.55 (6.71)	9.03 (4.83)	7.13 (5.81)
Generalized Anxiety Disorder (range 0-21), mean (SD)		3.56 (4.54)	2.30 (4.03)	6.49 (3.96)	3.15 (4.67)
Social Network Index, number of high-contact roles (range 0-12), mean (SD)		5.27 (1.26)	5.37 (1.09)	5.04 (1.28)	5.32 (1.43)
Social Network Index (number of people in the social network), mean (SD)		18.92 (8.38)	18.59 (7.91)	17.22 (7.15)	20.57 (9.49)
Social support score, mean (SD)		3.92 (0.65)	3.85 (0.61)	3.66 (0.49)	4.17 (0.70)
MCI^b^, n (%)		89 (20.6)	37 (19.1)	40 (39.2)	12 (8.8)
MCR^c^, n (%)		58 (13.4)	38 (19.6)	9 (8.8)	11 (8)

^a^BMH: Baby Memorial Hospital.

^b^MCI: mild cognitive impairment.

^c^MCR: motoric cognitive risk syndrome.

## Discussion

KES aims to understand the risk factors associated with MCR in the Kerala population. The initial phase of KES has already played a key role in defining and establishing risk factors and pathologies for MCR and MCI in India [13,18]. In this new project, our focus will shift to studying the role of apathy and TBI (risk factors) as well as cognitive reserve (protective factor) on the pathogenesis of cognitive decline and predementia syndromes in Kerala. Research on predementia syndromes such as MCR in LMIC such as India is scarce [89,90]. Emerging evidence emphasizes the interactions among ethnicity, genetics, environment, culture, and neurobiology in influencing manifestations of dementia [90]. We will make use of the interdisciplinary expertise of the KES team, local research infrastructure established since 2008, access to unique populations, and strong foundation of research to address our research questions focusing on predementia, especially MCR. This study also lays down a strong foundation of neuroepidemiological research in this region. The Kerala population is homogenous in terms of race, language, and culture. Hence, any observed findings should also apply to the broader Kerala older adult population.

Apathy is one of the prevalent and challenging behavioral symptoms associated with dementia [91]. Our recent study of a cohort based in the United States has shown apathy to be associated with incident MCR but not MCI [81]. Mild TBI is a novel risk factor that we address in this project. Although severe TBI-related injuries and deaths are common in India, mild TBI has not been established as a risk factor for late-life cognitive decline in Indian older adults. Neuroimaging studies to understand the association of TBI and other neuroimaging correlates (SVD, WMH, lacunes, microbleeds, gray matter volume, cortical thickness, and structural connectivity) with predementia syndromes including MCR have been scarce in this part of the world. Hence, this new KES project will provide new insights into the nature of cognitive decline in the growing older Indian population.

KES’s main strength is the extensive clinical data being collected from a unique population, which provides opportunities to carry out research focusing on cognitive decline as well as geroscience. This will also be the first as well as the largest organized study of predementia syndromes and cognitive decline in Kerala state. The research infrastructure established in past and current KES projects will be key to initiating future spin-off studies to understand other clinical and biological aspects of cognitive decline and aging. KES data can also be utilized to understand multiple other age-associated phenotypes and syndromes such as frailty and depression in the older adult population in this region.

The cross-sectional design of KES does not permit causal inferences. However, this will be overcome with the longitudinal study design, which will be initiated in this phase and expanded in the future. We have not proposed biological specimen collection. We will explore local collaborations with the goal of establishing a biorepository facility in Kozhikode, which will aid in conducting genetic and bioassays.

This KES project builds on the foundation of our past successful collaboration. Recruitment for the new KES project has started and is going well. We expect to achieve our recruitment goals and aims within the proposed project period. This KES project will be a major step toward understanding risk factors and neuroimaging correlates associated with MCR in Kerala and will help improve identification of older individuals at risk of dementia as well as guide future development of preventive strategies for dementia in India and elsewhere.

## Appendix

Multimedia Appendix 1 Peer-reviewer report from the National Institute on Aging and Fogarty International Center.

## Abbreviations

ACE: Addenbrooke Cognitive Examination
AES: Apathy Evaluation Scale
BISQ: Brain Injury Screening Questionnaire
BMH: Baby Memorial Hospital
DSM-IV: Diagnostic and Statistical Manual of Mental Disorders IV
ECG: electrocardiogram
FLAIR: fluid-attenuated inversion recovery
FOV: field of view
FSL: Functional Magnetic Resonance Imaging of the Brain Software Library
GAD: Generalized Anxiety Disorder
GDS: Geriatric Depression Scale
ICMR-NCTB: Indian Council of Medical Research-Neurocognitive Toolbox
IRB: institutional review board
KES: Kerala Einstein Study
LMEM: linear mixed effects model
LMIC: low and middle-income countries
MARS: Microbleed Anatomic Rating Scale
MCI: mild cognitive impairment
MCR: motoric cognitive risk syndrome
MRI: magnetic resonance imaging
RA: research assistant
SVD: small vessel disease
SWI: susceptibility-weighted imaging
TBI: traumatic brain injury
TE: echo time
TR: repetition time
TRACULA: Tracts Constrained by Underlying Anatomy
WMH: white matter hyperintensities

## Data Availability

The data set generated in this study is available from the corresponding author on reasonable request and with completion of the data user agreement.
