# Supplementary material for: Epidemiology of Motoric Cognitive Risk Syndrome in the Kerala Einstein Study: Protocol for a Prospective Cohort Study
Source: JMIR Res Protoc. 2023 Aug 17;12:e49933. doi: 10.2196/49933 (PMC10472178; doi:10.2196/49933)
Supplement: Multimedia Appendix 1 [file resprot_v12i1e49933_app1.pdf]

SUMMARY STATEMENT

(Privileged Communication)

PROGRAM CONTACT:
DALLAS ANDERSON
301-496-9350
andersda@nia.nih.gov

Release Date: **04/03/2019**
Revised Date:

Application Number: **2 R01 AG039330-07**

Principal Investigators (Listed Alphabetically):

MATHURANATH, PAVAGADA S
VERGHESE, JOE (Contact)

Applicant Organization: ALBERT EINSTEIN COLLEGE OF MEDICINE, INC

Review Group: **ZRG1 BDCN-N (55)**
Center for Scientific Review Special Emphasis Panel
PAR Panel: Fogarty Global Brain Disorders

Meeting Date: **03/12/2019**
Council: **MAY 2019**
Requested Start: **06/01/2019**

RFA/PA: **PAR18-835**
PCC: **3CPEPDA**
Dual IC(s): **TW, MH**

Project Title: **Kerala-Einstein Aging Study**

SRG Action: **Impact Score:22 Percentile:6 #**
Next Steps: **Visit https://grants.nih.gov/grants/next_steps.htm**
Human Subjects: 30-Human subjects involved - Certified, no SRG concerns
Animal Subjects: 10-No live vertebrate animals involved for competing appl.
Gender: 1A-Both genders, scientifically acceptable
Minority: 1A-Minorities and non-minorities, scientifically acceptable
Age: 1A-Both Children and Adults, scientifically acceptable

Project Year	Direct Costs Requested	Estimated Total Cost
7	455,002	681,862
8	444,007	665,385
9	427,507	640,658
10	432,507	648,151
11	426,907	639,759
TOTAL	2,185,930	3,275,814

2R01AG039330-07 VERGHESE, JOE

RESUME AND SUMMARY OF DISCUSSION: This project seeks to identify risk factors for pre-dementia using Motoric Cognitive Risk syndrome (MCR) measures in urban and rural seniors in the Indian state of Kerala. Dr. Verghese pioneered the concept of MCR, a new pre-dementia disorder characterized by cognitive complaints and slow gait. The investigative team is highly productive and has published together extensively. Strengths include the prior success in capacity building including advanced neuroimaging and a bio-repository, expansion of capacity to rural and ex-urban LMIC setting, broader definition of cognitive impairments that considers mild TBI and vascular dementia, and neuroimaging of a subset of subjects. There were minor concerns regarding transition from a 1.5T to a 3T scanner. There was discussion of the failure of the investigators to consider nutritional differences in rural populations with consumption of cassava. Overall, the panel was enthusiastically supportive of this study that will inform preventative interventions for Alzheimer's disease and related dementias by an outstanding team of investigators.

DESCRIPTION (provided by applicant): The Kerala-Einstein Study (KES) proposes to build on the successful activities in our previous funding period to conduct high quality and impact research into Alzheimer's disease and related dementias in the southern Indian state of Kerala. The KES team successfully established clinical research center in Kerala, trained junior investigators, instituted uniform study procedures, developed culture-fair cognitive tests, built databases, created research neuroimaging protocols, established a biorepository, and had multiple publications. In this renewal, our focus is on risk factors and brain substrates of pre-dementia syndromes, especially Motoric Cognitive Risk syndrome (MCR). MCR is a recent and innovative concept proposed by Dr. Verghese and colleagues and validated in multiple countries including low and middle-income countries (LMIC). MCR is a pre-dementia syndrome characterized by presence of cognitive complaints and slow gait. MCR predicts risk of both Alzheimer's disease and vascular dementia even after accounting for overlap with Mild Cognitive Impairment syndrome (MCI). Unlike MCI, complex cognitive tests or assays are not needed to diagnose MCR, increasing its clinical utility in resource poor LMIC settings. Aim 1. Examine clinical risk factors for pre-dementia syndromes (MCR and MCI) in 1000 Kerala seniors. We identified potentially modifiable risk factors (depression, sedentariness and obesity) for MCR in developed countries, but their association with MCR in LMIC is unknown. In addition, we will study novel risk factors for pre- dementia syndromes in India; apathy and mild traumatic brain injury, and cognitive reserve. Aim 2. Explore risk factors for pre-dementia syndromes in 400 rural and 600 urban Kerala seniors. There is a paucity of cognitive studies in rural seniors globally; our urban and rural sites in Kerala will provide a valuable opportunity to compare and contrast risk factors for MCR and MCI. In this renewal, we propose to establish a new research center in a rural site. Aim 3. Establish brain pathologies and substrates of MCR in Kerala seniors. Frontal lacunes and frontal gray matter thinning were associated with MCR in KES, implicating both vascular and neurodegenerative pathologies. Small vessel disease is associated with gray matter atrophy. We propose to test mechanistic hypotheses linking these two pathologies to MCR syndrome in 400 individuals with neuroimaging. We will continue to examine the neurobiological basis of pre-dementia syndromes in the KES biorepository that contains genetic samples of over 500 clinically phenotyped patients (normal, MCI and dementia). Our studies have the potential to continue to have a major impact in continuing to build sustainable research capacity in India, foster research training in India and USA and elucidate the pathogenesis of dementia.

PUBLIC HEALTH RELEVANCE: The Kerala-Einstein Study (KES) proposes to build on the successful research collaboration in our last funding period to conduct high quality and impactful research into Alzheimer's disease and related dementias in the Indian state of Kerala. In this renewal, we propose to target risk factors and brain substrates of pre-dementia syndromes, especially the recently Motoric Cognitive Risk syndrome (MCR). MCR is characterized by cognitive complaints and slow gait and identifies older adults at high risk for both Alzheimer's disease and vascular dementia. Our studies have the potential to have a major impact in building sustainable research capacity in India, foster research

training in India and USA, elucidate the pathogenesis of dementia, and provide insights into modifiable risk factors for dementia; ultimately leading to development of treatment and prevention strategies that are applicable worldwide.

CRITIQUE 1

Significance: 2
Investigator(s): 1
Innovation: 3
Approach: 2
Environment: 1

Overall Impact: This is a renewal application of a project originally funded 8 years ago, to study Alzheimer's disease and related dementias in the southern Indian state of Kerala. The Kerala-Einstein Study (KES) was initially established with a Fogarty R21 in 2008. The investigators have been very productive during the previous funding cycle, and an impressive infrastructure is in place, including the ability to do advanced neuroimaging and collect and bank biological fluids. During the next funding cycle, the proposal will focus on pre-dementia syndromes, particularly the Motoric Cognitive Risk (MCR) syndrome, which is not specific to amyloid pathologies but also incorporates risk from other pathologies such as vascular dementia and mild TBI. Dr. Verghese has been a leader in studies of MCR in the US, and this proposal will extend these studies into an LMIC setting, where the distribution of dementia-related pathologies may be different.

1. Significance

Strengths

- Significance is high. Dementia is rapidly becoming a major public health burden in LMICs, coincident with the decline in infectious diseases and other conditions that cause premature death.
- The MCR syndrome is a readily assessed condition which has clear clinical relevance and can be directly targeted with preventative interventions.

Weaknesses

- The KES does not incorporate several important tools which have made major impacts in disease in ADRD in developed countries, such as the use of genomics and protein biomarkers. Given the circumstances and unique genetics in southern India, there are likely to be important insights that arise from such studies.

2. Investigator(s):

Strengths

- The investigators are outstanding. Dr. Verghese graduated from medical school in India and trained in neurology in England and the US. He is a senior investigator who has worked his entire career at Albert Einstein College of Medicine and is currently chief of the divisions of cognitive and motor aging in the Departments of Neurology and Geriatrics. He has been a pioneer on the concept of Motoric Cognitive Risk and has many important papers and grants on this issue.
- The other members of the investigative team are also highly productive investigators, both at Albert Einstein and in India. Most have published extensively as a team and have experience working together.
- There are well-established connections with local investigators in India.

3. Innovation:

Strengths

- Innovation is modest. The MCR syndrome has been widely studied throughout the world. Contribution from vascular pathologies or other pathologies such as mild TBI are likely to be important and likely to be enriched in LMICs.
- The populations studied in the KES will include a substantial number of rural dwellers, which is likely to provide novel insights regarding risk factors.
- The focus on TBI is moderately innovative. While TBI has been long-recognized as a risk factor for late life dementia, it has rarely been directly studied as is proposed here. A local adaptation of a Brain Injury Screening Questionnaire will be used.

Weaknesses

- No use is made of valuable Biorepository resources (we are told that genetic samples from >500 carefully phenotypes individuals exist). While this is a relatively modest number by GWAS standards, it is reasonable for candidate gene studies, including analysis of polygenic risk scores.
- Additional focus on the contribution of diet to dementia risk is warranted. In particular, southern India and Kerala are areas where cassava is widely consumed.

4. Approach:

Strengths

- The KES has been very successful over the prior funding cycles in establishing valuable research infrastructure, including the capability for advanced neuroimaging and collection of biological samples.
- 18 publications have come from the prior funding cycle.
- Recruitment will be from urban, rural, and exurban settings. 800 new participants will be recruited. MCR, MCI, self-reported history of mild TBI, and self-reported apathy/depression symptoms will be assessed. A focused neuropsychometric battery will also be administered.
- Neuroimaging will be carried out in a subset (n=220), initially on a 1.5 T scanner, with plans to transition to a 3 T scanner. Volume and cortical thickness will be quantified, as well as white matter integrity using DTI.
- Follow-up 12 months after the initial visit will be done on all rural participants and all those who completed an MRI.

Weaknesses

- Assessment of vascular risk factors, although a key goal of this application, is superficial.

5. Environment:

Strengths

- The KES study is well-established, and an outstanding platform for conducting the proposed study.
- Kerala State is among more urbanized and the better educated regions of India, and by some development indices is on par with some HICs. The life expectance in Kerala state (73.5 years is substantially higher than in India as a whole (61.7 years).

Capacity Building:

- Excellent capacity building plans are included. This comes on top of substantial past success. Additional ex-urban and rural sites are added, which were not part of the project in prior funding cycles.

Study Timeline:

Strengths

- Gannt chart is included.

Protections for Human Subjects:

- Acceptable Risks and/or Adequate Protections

Data and Safety Monitoring Plan (Applicable for Clinical Trials Only):

- Not Applicable (No Clinical Trials)

Inclusion of Women, Minorities and Children:

- Sex/Gender: Distribution justified scientifically
- Race/Ethnicity: Distribution justified scientifically
- For NIH-Defined Phase III trials, Plans for valid design and analysis: Not applicable
- Inclusion/Exclusion of Children under 18: Excluding ages <18; justified scientifically

Vertebrate Animals:

- Not Applicable (No Vertebrate Animals)

Biohazards:

- Not Applicable (No Biohazards)

Applications from Foreign Organizations:

- Justified

Select Agents:

- Not Applicable (No Select Agents)

Resource Sharing Plans:

- Acceptable

Authentication of Key Biological and/or Chemical Resources:

- Not Applicable (No Relevant Resources)

Budget and Period of Support:

Recommend as Requested

CRITIQUE 2

Significance: 3
Investigator(s): 1
Innovation: 3
Approach: 3
Environment: 3

Overall Impact: The overall goal of the study is to identify risk factors related to MCR, a disorder that has been shown to be a physiological state that precipitates the pathogenesis of AD and related dementias. As such, this proposal is highly significant. The investigators complement each other, and their expertise ensures that the project will be completed successfully, as was the initial study. The approach is sound, but this project tends to suffer from a lack of innovation. As in, this proposal relies mostly upon prior evidence and to expand upon those results. While MCR is an innovative concept, much of the innovation is derived from the initial study and other ongoing projects, rather than from novel concepts and experimental designs/approaches.

1. Significance:

Strengths

- The overall goal of the study is to build on research that was previously conducted using the same funding mechanism that focused on Alzheimer's disease (AD) and related dementias in the Indian state of Kerala. The collaborative efforts of the Indo-US team resulted in establishing both a bio-repository as well as a clinical research center in Kozhikode, Kerala as well as several other accomplishments, including implementing neuroimaging protocols and building research databases. The investigators now seek to build upon those results by focusing on risk factors and brain substrates that contribute to the onset of Motoric Cognitive Risk syndrome (MCR), a new pre-dementia disorder identified by these investigators that is characterized by cognitive complaints and slow gait. Risk factors for MCR in high income countries include depression, sedentariness, and obesity. The aim of this study is to identify modifiable risk factors for MCR in low-and-middle income countries (LMICs), which are believed to include apathy and traumatic brain injury. This proposal also seeks to make a distinction between MCR and mild cognitive impairment (MCI), and further, to identify neuropathologies associated with MCR. Given the recently identified disease burden of MCR, and the disease burden of AD this proposal is highly significant.

Weaknesses

- No weaknesses were noted.

2. Investigator(s):

Strengths

- Joe Verghese, MBBS, MRCPI is the Principal Investigator. He is currently a Professor of Neurology and Medicine at Albert Einstein College of Medicine. Dr. Verghese has previously served as the Principal Investigator of the initial Kerala-Einstein Aging Study and, as a result, has extensive experience with epidemiological techniques, clinical trial experiences, experimental test development, neuroimaging, and investigations of biomarkers as well as genetic markers in the context of aging and dementia. He is joined by and Dr. Pavagada

Sivasankara Mathuranath, who will serve as co-Principal Investigator of the study. Dr. Mathuranath is an Additional Professor at the National Institute of Mental Health and Neurosciences in Bangalore, India, and a visiting professor at Albert Einstein College of Medicine. Other co-investigators are Dr. Helena Blumen, Dr. Cuiling Wang, and Dr. Mirnova Ceide, all of Albert Einstein College of Medicine. They are also joined by Dr. Anne Ambrose. Dr. Anne Ambrose is the Co-Director of Department of Physical Medicine at the Winifred Masterson Burke Medical Research Institute. Drs. Ambrose and Mathuranath have both previously participated in the Kerala-Einstein Aging Study and their varied sets of expertise complement each other, and their synergistic efforts ensure that the renewal will be just as successful as the initial study.

Weaknesses

- No weaknesses were noted.

3. Innovation:

Strengths

- MCR is an innovative concept, proposed by Dr. Verghese and his team. The expansion of MCR as a concept has led to the need to identify risk factors for the disorder, which have been determined to be TBI, apathy, and cognitive reserve. The inclusion of these as risk factors is innovative, and the emphasis on neuroimaging as diagnosis is innovative in rural environments such as this one. Further, the diagnostic approach to identifying MCR, particularly the assessment of gait repurposing the GAITRite System to diagnose pre-dementia and the addition of an algorithm to diagnose patients, is innovative.

Weaknesses

- While this proposal is novel overall, the investigators have placed most of the emphasis on their prior accomplishments and on how they extend the innovative aspects of the initial study and various other studies that Dr. Verghese is involved in to the renewal. As a result, this study is not entirely novel on its own, but rather in the context of the other projects.

4. Approach

Strengths

- The investigators intend to comprehensively evaluate the contribution of risk factors to the occurrence of MCR, and their project is designed in such a way that will enable them to do so. The data is scarce, and specifically, the neuroimaging data is severely lacking. This project will rectify these and enhance our understanding of dementia and pre-dementia in rural and urban communities in LMICs.

Weaknesses

- No weaknesses were noted.

5. Environment:

Strengths

- The primary sites for this study are Albert Einstein College of Medicine (AECOM), Baby Memorial Hospital in Kozhikode, Kerala, India, the National Institute of Mental Health and Neuroscience in Bangalore, India, and Meitra Hospital in Edakkad, Kerala, India. All of these sites are fully equipped with the necessary equipment to execute the studies. At AECOM, Dr. Verghese's lab is fully equipped and further supported by the Department of Cognitive and Motor Aging, which provides him with a clinical research team. Both Baby Memorial Hospital

and Meitra Hospital have neuroimaging facilities, including an MRI scanner, and fully operational labs that will enable the investigators to execute their studies and to properly examine patients to look for MCR. The National Institute of Mental Health and Neuroscience provides support to Dr. Mathuranath. Additionally, the investigators have included Kakkodi village as their rural environment. While there isn't any hospital in Kakkodi, the site offers investigators the unique opportunity to assess MCR prevalence and incidence in rural settings. The project will greatly benefit from the unique features of the multiple environments.

Weaknesses

- No weaknesses were noted.

Capacity Building:

- The research strategy does contain appropriate plans to address the capacity building component of the RFA.

Study Timeline:

Strengths

- The study timeline is appropriate for the proposal.

Weaknesses

- No weaknesses were noted.

Protections for Human Subjects:

- Acceptable Risks and/or Adequate Protections
- The risks for human subjects proposed here are acceptable and the protections are adequate.

Data and Safety Monitoring Plan (Applicable for Clinical Trials Only):

- Acceptable
- The data and safety monitoring plan in this application is acceptable.

Inclusion of Women, Minorities and Children:

- Sex/Gender: Distribution justified scientifically
- Race/Ethnicity: Distribution justified scientifically
- For NIH-Defined Phase III trials, Plans for valid design and analysis: Not applicable
- Inclusion/Exclusion of Children under 18: Excluding ages <18; justified scientifically

Vertebrate Animals:

- Not Applicable (No Vertebrate Animals)

Biohazards:

- Not Applicable (No Biohazards)

Renewal:

- This application is a renewal of previously funded grant. The investigators seek to build on their prior findings by refocusing on risk factors and brain substrates for MCR in Kerala seniors.

Budget and Period of Support:

Recommend as Requested

CRITIQUE 3

Significance: 2
Investigator(s): 1
Innovation: 2
Approach: 2
Environment: 1

Overall Impact: This is an excellent renewal proposal by a well-established, highly productive team of investigators to study MCR, MCI and related risk factors in both an urban and rural LMIC setting. These studies are impactful and innovative with strong scientific premise. Only minor concerns are noted.

1. Significance:

Strengths

- Study of MCR and risk factors in LCMI is significant.
- Scientific premise is supported by preliminary data and literature.
- The consideration of another predementia syndrome in addition to MCI is highly significant.
- The collection of data from a rural sample is also highly significant.
- TBI appears to play a unique role in cognitive dysfunction risk in LMIC.

Weaknesses

- None noted

2. Investigator(s):

Strengths

- This is an excellent study team with experience to carry out the studies as proposed and a track record of work together.
- The schematic in the budget justification is incredibly useful in determining how these investigators will interact.
- The team has been very productive during the previous grant period.

Weaknesses

- None noted

3. Innovation:

Strengths

- The study of MCR and its risk factors in LMIC is highly innovative.
- The study of TBI as it relates to cognitive risk specific to LMIC is also highly innovative.

Weaknesses

- The proposal of a mechanism related to vascular disease is not novel.

4. Approach:

Strengths

- The description of the interactions between the study team are fantastic.
- The description of the study sites is also very helpful.
- The existing infrastructure suggest a high likelihood for success.
- The potential for longitudinal imaging in an LMIC is exciting and important.

Weaknesses

- Are there differences in exposures in the urban and rural populations (i.e., pesticides, pollutants) that could bias any potential differences observed.
- Further, wouldn't one expect differences in PA between rural and urban residents just given lifestyle differences.
- What QC methods will be performed on neuroimaging data, motion often increases with cognitive impairment and could potentially systematically bias the data.

5. Environment:

Strengths

- Environments are excellent.

Weaknesses

- None noted.

Capacity Building:

- Significant opportunity for capacity building with exploration of new prodromal cognitive syndrome and expansion of neuroimaging and genetics capacities.

Protections for Human Subjects:

- Acceptable Risks and/or Adequate Protections

Data and Safety Monitoring Plan (Applicable for Clinical Trials Only):

- Not Applicable (No Clinical Trials)

Inclusion of Women, Minorities and Children:

- Sex/Gender: Distribution justified scientifically
- Race/Ethnicity: Distribution justified scientifically
- For NIH-Defined Phase III trials, Plans for valid design and analysis:
- Inclusion/Exclusion of Children under 18: Excluding ages <18; justified scientifically

Vertebrate Animals:

- Not Applicable (No Vertebrate Animals)

Biohazards:

- Not Applicable (No Biohazards)

Applications from Foreign Organizations:

- Justified

Select Agents:

- Not Applicable (No Select Agents)

Resource Sharing Plans:

- Acceptable

Authentication of Key Biological and/or Chemical Resources:

- Not Applicable (No Relevant Resources)

Budget and Period of Support:

Recommend as Requested

THE FOLLOWING SECTIONS WERE PREPARED BY THE SCIENTIFIC REVIEW OFFICER TO SUMMARIZE THE OUTCOME OF DISCUSSIONS OF THE REVIEW COMMITTEE, OR REVIEWERS' WRITTEN CRITIQUES, ON THE FOLLOWING ISSUES:

PROTECTION OF HUMAN SUBJECTS: ACCEPTABLE

INCLUSION OF WOMEN PLAN: ACCEPTABLE

INCLUSION OF MINORITIES PLAN: ACCEPTABLE

INCLUSION OF CHILDREN PLAN: ACCEPTABLE

COMMITTEE BUDGET RECOMMENDATIONS: The budget was recommended as requested.

Footnotes for 2 R01 AG039330-07; PI Name: VERGHESE, JOE

Ad hoc or special section application percentiled against "Total CSR" base.

NIH has modified its policy regarding the receipt of resubmissions (amended applications). See Guide Notice NOT-OD-14-074 at http://grants.nih.gov/grants/guide/notice-files/NOT-OD-14-074.html. The impact/priority score is calculated after discussion of an application by averaging the overall scores (1-9) given by all voting reviewers on the committee and multiplying by 10. The criterion scores are submitted prior to the meeting by the individual reviewers assigned to an application, and are not discussed specifically at the review meeting

or calculated into the overall impact score. Some applications also receive a percentile ranking. For details on the review process, see http://grants.nih.gov/grants/peer_review_process.htm#scoring.

MEETING ROSTER

Center for Scientific Review Special Emphasis Panel
CENTER FOR SCIENTIFIC REVIEW
PAR Panel: Fogarty Global Brain Disorders

ZRG1 BDCN-N (55)
03/12/2019 - 03/13/2019

Notice of NIH Policy to All Applicants: Meeting rosters are provided for information purposes only. Applicant investigators and institutional officials must not communicate directly with study section members about an application before or after the review. Failure to observe this policy will create a serious breach of integrity in the peer review process, and may lead to actions outlined in NOT-OD-14-073 at https://grants.nih.gov/grants/guide/notice-files/NOT-OD-14-073.html and NOT-OD-15-106 at https://grants.nih.gov/grants/guide/notice-files/NOT-OD-15-106.html, including removal of the application from immediate review.

CHAIRPERSON(S)

FURIE, KAREN L, MD
PROFESSOR AND CHAIR
DEPARTMENT OF NEUROLOGY
THE WARREN ALPERT MEDICAL SCHOOL
BROWN UNIVERSITY
PROVIDENCE, RI 02903

MEMBERS

BALACHOVA, TATIANA N, PHD
PROFESSOR
DEPARTMENT OF PEDIATRICS
CHILD STUDY CENTER
THE UNIVERSITY OF OKLAHOMA HEALTH SCIENCES CENTER
OKLAHOMA CITY, OK 73117

BARR, DANA B, PHD
PROFESSOR
DEPARTMENT OF ENVIRONMENTAL HEALTH
ROLLINS SCHOOL OF PUBLIC HEALTH
EMORY UNIVERSITY
ATLANTA, GA 30322

BENDLIN, BARBARA B, PHD
ASSOCIATE PROFESSOR
SCHOOL OF MEDICINE AND PUBLIC HEALTH
WISCONSIN ALZHEIMER'S DISEASE RESEARCH CENTER
UNIVERSITY OF WISCONSIN
MADISON, WI 53792

BERLIN, LISA J, PHD
ASSOCIATE PROFESSOR
SCHOOL OF SOCIAL WORK
UNIVERSITY OF MARYLAND
BALTIMORE, MD 21201

BUCKLEY, PETER F, MD
DEAN
SCHOOL OF MEDICINE
VIRGINIA COMMONWEALTH UNIVERSITY
RICHMOND, VA 23298

BUTLER, LISA M, PHD
ASSOCIATE RESEARCH PROFESSOR
INSTITUTE FOR COLLABORATION ON HEALTH, INTERVENTION, AND POLICY
UNIVERSITY OF CONNECTICUT
STORRS, CT 06269

CALDWELL, KAREN L, PHD
PROFESSOR
DEPARTMENT OF HUMAN DEVELOPMENT AND PSYCHOLOGICAL COUNSELING
REICH COLLEGE OF EDUCATION
APPALACHIAN STATE UNIVERSITY
BOONE, NC 28608

CHEN, AIMIN, MD, PHD
PROFESSOR
DEPARTMENT OF ENVIRONMENTAL HEALTH
COLLEGE OF MEDICINE
UNIVERSITY OF CINCINNATI
CINCINNATI, OH 45267

COHEN, ANN D, PHD
ASSISTANT PROFESSOR
DEPARTMENT OF PSYCHIATRY
UNIVERSITY OF PITTSBURGH
PITTSBURH, PA 15213

CRNIC, KEITH A, PHD
PROFESSOR
DEPARTMENT OF PSYCHOLOGY
ARIZONA STATE UNIVERSITY
TEMPE, AZ 85287

DESRUISSEAUX, MAHALIA S, MD
ASSISTANT PROFESSOR
DEPARTMENT OF INTERNAL MEDICINE
SCHOOL OF MEDICINE
YALE UNIVERSITY
NEW HAVEN, CT 06510

DIAZ-ARRASTIA, RAMON, MD, PHD
PRESIDENTIAL PROFESSOR OF NEUROLOGY
PENN'S PERELMAN SCHOOL OF MEDICINE
UNIVERSITY OF PENNSYLVANIA
PHILADELPHIA, PA 19104

DONAHUE, MANUS J, PHD
ASSOCIATE PROFESSOR
DEPARTMENT OF MEDICAL IMAGING
VANDERBILT UNIVERSITY
NASHVILLE, TN 37232

DOZIER, MARY, PHD
PROFESSOR
DEPARTMENT OF PSYCHOLOGY
UNIVERSITY OF DELAWARE
NEWARK, DE 19716

FACTOR-LITVAK, PAM R, PHD
PROFESSOR AND ASSOCIATE DEAN
DEPARTMENT OF EPIDEMIOLOGY
MAILMAN SCHOOL OF PUBLIC HEALTH
COLUMBIA UNIVERSITY
NEW YORK, NY 10032

FIEDLER, NANCY L, PHD
PROFESSOR AND DEPUTY DIRECTOR
DEPARTMENT OF ENVIRONMENTAL AND
OCCUPATIONAL MEDICINE
SCHOOL OF PUBLIC HEALTH
RUTGERS UNIVERSITY
PISCATAWAY, NJ 08854

FRIM, DAVID M, MD, PHD
PROFESSOR AND CHIEF
DEPARTMENT OF PEDIATRICS
SECTION OF NEUROSURGERY
UNIVERSITY OF CHICAGO
CHICAGO, IL 60637

GALANOPOULOU, ARISTEA S, MD, PHD
PROFESSOR
DEPARTMENTS OF NEUROLOGY
AND NEUROSCIENCE
ALBERT EINSTEIN COLLEGE OF MEDICINE
BRONX, NY 10461

GERMAN, DWIGHT C, PHD
PROFESSOR
DEPARTMENT OF PSYCHIATRY
SOUTHWESTERN MEDICAL SCHOOL
UNIVERSITY OF TEXAS
DALLAS, TX 75235

GORELICK, PHILIP B, MD
PROFESSOR
DEPARTMENT OF NEUROLOGY AND REHABILITATION
UNIVERSITY OF ILLINOIS COLLEGE OF MEDICINE
CHICAGO, IL 60612

GUR, RUBEN C, PHD
PROFESSOR
DEPARTMENT OF PSYCHIATRY
UNIVERSITY OF PENNSYLVANIA
PHILADELPHIA, PA 19104

HE, SHUMAN, PHD, MD
ASSOCIATE PROFESSOR
DEPARTMENT OF OTOLARYNGOLOGY
THE OHIO STATE UNIVERSITY
COLUMBUS, OH 43212

HERMANN, BRUCE P, PHD
PROFESSOR
DEPARTMENT OF NEUROLOGY
UNIVERSITY OF WISCONSIN
MADISON, WI 53792

HUANG, KENG-YEN, PHD
ASSOCIATE PROFESSOR
DEPATMENT OF POPULATION HEALTH
CENTER FOR EARLY CHILDHOOD HEALTH AND
DEVELOPMENT
NEW YORK UNIVERSITY LANGONE HEALTH
NEW YORK, NY 10016

JASPAN, HEATHER B, MD, PHD
ASSISTANT PROFESSOR
DEPARTMENT OF PEDIATRICS AND GLOBAL HEALTH
SEATTLE CHILDREN'S HOSPITAL
SEATTLE, WA 98101

KEEP, RICHARD F, PHD
PROFESSOR
DEPARTMENT OF NEUROSURGERY
UNIVERSITY OF MICHIGAN
ANN ARBOR, MI 48109

KHANNA, RAJESH, PHD
PROFESSOR
DEPARTMENT OF PHARMACOLOGY
UNIVERSITY OF ARIZONA
TUCSON, AZ 85724

LAJINESS-O'NEILL, RENEE R, PHD
PROFESSOR
DEPARTMENT OF PSYCHOLOGY
EASTERN MICHIGAN UNIVERSITY
YPSILANTI, MI 48197

MARSH, ELISABETH B, MD
ASSOCIATE PROFESSOR
DEPARTMENT OF NEUROLOGY
JOHNS HOPKINS UNIVERSITY
SCHOOL OF MEDICINE
BALTIMORE, MD 21287

MASELKO, JOANNA, SCD
ASSOCIATE PROFESSOR
DEPARTMENT OF EPIDEMIOLOGY
GILLINGS SCHOOL OF GLOBAL PUBLIC HEALTH
UNIVERSITY OF NORTH CAROLINA
CHAPEL HILL, NC 7599

MATHALON, DANIEL H, MD, PHD
PROFESSOR
DEPARTMENT OF PSYCHIATRY
SCHOOL OF MEDICINE
UNIVERSITY OF CALIFORNIA, SAN FRANCISCO
SAN FRANCISCO, CA 94121

MAZUMDAR, MAITREYI, MD
ASSOCIATE PROFESSOR
DEPARTMENT OF NEUROLOGY
BOSTON CHILDREN'S HOSPITAL
HARVARD MEDICAL SCHOOL
BOSTON, MA 02115

MEYER, ANA-CLAIRE L, MD
SENIOR CLINICAL ADVISOR
MEDICAL RESEARCH AND MATERIEL COMMAND
UNITED STATE ARMY
FREDERICK, MD 21705

MEYER, DAWN M, PHD
ASSOCIATE PROFESSOR
DEPARTMENT OF NEUROSCIENCES
SCHOOL OF MEDICINE
UNIVERSITY OF CALIFORNIA, SAN DIEGO
LA JOLLA, CA 92093

O'DONNELL, JAMES M, PHD
DEAN AND PROFESSOR
DEPARTMENT OF PHARMACEUTICAL SCIENCES
UNIVERSITY AT BUFFALO
THE STATE UNIVERSITY OF NEW YORK
BUFFALO, NY 14214

PASINETTI, GIULIO M, MD, PHD
SENIOR RESEARCH CAREER SCIENTIST
THE SAUNDERS FAMILY CHAIR
CENTER FOR MOLECULAR INTEGRATIVE
NEURORESILIENCE
ICAHN SCHOOL OF MEDICINE AT MOUNT SINAI
JAMES J. PETERS VETERANS AFFAIRS MEDICAL CENTER
BRONX, NY 10468

RAMADOSS, JAYANTH, PHD
ASSOCIATE PROFESSOR
DEPARTMENT OF PHYSIOLOGY AND PHARMACOLOGY
COLLEGE OF VETERINARY MEDICINE AND
BIOMEDICAL SCIENCES
TEXAS A&M UNIVERSITY
COLLEGE STATION , TX 77483

ROBINSON, DELBERT G, MD
PROFESSOR
DEPARTMENT OF RESEARCH
ZUCKER HILLSIDE HOSPITAL
NORTH SHORE-LONG ISLAND JEWISH HEALTH SYSTEM
GLEN OAKS, NY 11004

ROHLMAN, DIANE S, PHD
ASSOCIATE PROFESSOR
OCCUPATIONAL AND ENVIRONMENTAL HEALTH
UNIVERSITY OF IOWA
IOWA CITY, IA 52242

RORDEN, CHRISTOPHER, PHD
PROFESSOR
DEPARTMENT OF PSYCHOLOGY
ENDOWED CHAIR OF NEUROIMAGING
UNIVERSITY OF SOUTH CAROLINA
COLUMBIA, SC 29208

SABA, LAURA M, PHD
ASSOCIATE PROFESSOR
DEPARTMENT OF PHARMACEUTICAL SCIENCES
SKAGGS SCHOOL OF PHARMACY AND
PHARMACEUTICAL SCIENCE
UNIVERSITY OF COLORADO DENVER
PARKER, CO 80134

SINGER, ELYSE J, MD
ASSOCIATE CLINICAL PROFESSOR
DEPARTMENT OF NEUROLOGY
UNIVERSITY OF CALIFORNIA LOS ANGELES
LOS ANGELES, CA 90025

SMITH, GWENN S, PHD
PROFESSOR
DEPARTMENT OF PSYCHIATRY AND BEHAVIORAL
SCIENCES
JOHNS HOPKINS UNIVERSITY SCHOOL OF MEDICINE
BALTIMORE, MD 21224

SNYDER, EVAN Y, MD, PHD
PROFESSOR
HUMAN GENETICS PROGRAM
DIRECTOR CENTER STEM CELLS AND
REGENERATIVE MEDICINE
SANFORD BURNHAM PREBYS MEDICAL DISCOVERY
INSTITUTE
LA JOLLA, CA 92037

SPENCER, PETER S, PHD
PROFESSOR
DEPARTMENT OF NEUROLOGY, SCHOOL OF MEDICINE
OREGON INSTITUTE OF OCCUPATIONAL HEALTH SCIENCES
OREGON HEALTH AND SCIENCES UNIVERSITY
PORTLAND, OR 97239

SULLIVAN, JAN G, MD
PROFESSOR
SCHOOL OF MEDICINE
UNIVERSITYOF CALIFORNIA, RIVERSIDE
RIVERSIDE, CA 92521

VULCHANOVA, LYUDMILA H, PHD
ASSOCIATE PROFESSOR
DEPARTMENT OF NEUROSCIENCE
UNIVERSITY OF MINNESOTA
ST PAUL, MN 55116

WHEELER, ANNE C, PHD
RESEARCH PUBLIC HEALTH ANALYST
RESEARCH TRIANGLE INSTITUTE INTERNATIONAL
RESEARCH TRIANGLE INSTITUTE
RESEARCH TRIANGLE PARK, NC 27709

YANG, LAWRENCE H, PHD
ASSOCIATE PROFESSOR
DEPARTMENT OF EPIDEMIOLOGY
MAILMAN SCHOOL OF PUBLIC HEALTH
COLUMBIA UNIVERSITY
NEW YORK, NY 10032

YIANNOUTSOS, CONSTANTIN T, PHD
PROFESSOR
DEPARTMENT OF BIOSTATISTICS
INDIANA UNIVERSITY SCHOOL OF PUBLIC HEALTH
INDIANAPOLIS, IN 46202

ZHI, DEGUI, PHD
ASSOCIATE PROFESSOR
SCHOOL OF BIOMEDICAL INFORMATICS
THE UNIVERSITY OF TEXAS HEALTH SCIENCE
CENTER AT HOUSTON
HOUSTON, TX 77030

SCIENTIFIC REVIEW OFFICER

DRGONOVA, JANA, PHD
SCIENTIFIC REVIEW OFFICER
CENTER FOR SCIENTIFIC REVIEW
NATIONAL INSTITUTES OF HEALTH
BETHESDA, MD 20817

NADI, SUZAN, PHD
SCIENTIFIC REVIEW OFFICER
CENTER FOR SCIENTIFIC REVIEW
NATIONAL INSTITUTES OF HEALTH
BETHESDA, MD 20892

EXTRAMURAL SUPPORT ASSISTANT

KIRKPATRICK, JOEL D
LEAD EXTRAMURAL SUPPORT ASSISTANT
CENTER FOR SCIENTIFIC REVIEW
NATIONAL INSTITUTES OF HEALTH
BETHESDA, MD 20892

Consultants are required to absent themselves from the room during the review of any application if their presence would constitute or appear to constitute a conflict of interest.
